# A novel alphaproteobacterial ectosymbiont promotes the growth of the hydrocarbon-rich green alga *Botryococcus braunii*

**DOI:** 10.1038/srep10467

**Published:** 2015-07-01

**Authors:** Yuuhiko Tanabe, Yusuke Okazaki, Masaki Yoshida, Hiroshi Matsuura, Atsushi Kai, Takashi Shiratori, Ken-ichiro Ishida, Shin-ichi Nakano, Makoto M. Watanabe

**Affiliations:** 1 Faculty of Life & Environmental Sciences, University of Tsukuba; 2 Center for Ecological Research, Kyoto University; 3 Graduate School of Life & Environmental Sciences, University of Tsukuba.

## Abstract

*Botryococcus braunii* is a colony-forming green alga that accumulates large amounts of liquid hydrocarbons within the colony. The utilization of *B. braunii* for biofuel production is however hindered by its low biomass productivity. Here we describe a novel bacterial ectosymbiont (BOTRYCO-2) that confers higher biomass productivity to *B. braunii*. 16S rDNA analysis indicated that the sequence of BOTRYCO-2 shows low similarity (<90%) to cultured bacterial species and located BOTRYCO-2 within a phylogenetic lineage consisting of uncultured alphaproteobacterial clones. Fluorescence *in situ* hybridization (FISH) studies and transmission electric microscopy indicated that BOTRYCO-2 is closely associated with *B. braunii* colonies. Interestingly, FISH analysis of a water bloom sample also found BOTRYCO-2 bacteria in close association with cyanobacterium *Microcystis aeruginosa* colonies, suggesting that BOTRYCO-2 relatives have high affinity to phytoplankton colonies. A PCR survey of algal bloom samples revealed that the BOTRYCO-2 lineage is commonly found in *Microcystis* associated blooms. Growth experiments indicated that *B. braunii* Ba10 can grow faster and has a higher biomass (1.8-fold) and hydrocarbon (1.5-fold) yield in the presence of BOTRYCO-2. Additionally, BOTRYCO-2 conferred a higher biomass yield to BOT-22, one of the fastest growing strains of *B. braunii*. We propose the species name ‘*Candidatus* Phycosocius bacilliformis’ for BOTRYCO-2.

Traditionally, phytoplankton population dynamics in nature were considered to be mainly controlled by abiotic factors such as light, temperature, and nutrient availability, together with predatory factors^1^. However, recent studies have revealed the substantial contribution of bacterial associates to the rise and fall of phytoplankton populations^2^,^3^,^4^, highlighted by the algal–bacterial symbiosis in the oceans 5,6. Bacterial symbionts of marine microalgae are phylogenetically diverse. Several species in the *Roseobacter* clade (*Alphaproteobacteria*) are mutualistic partners of marine dinoflagellates and coccolithophores^6^,^7^; and *Marinobacter* spp. (*Gammaproteobacteria*) have a mutualistic relationship with marine dinoflagellates, coccolithophores, and diatoms^8^. In contrast, algal–bacterial symbiotic relationships in freshwater environments and their ecological significance have been poorly investigated. This scientific ignorance is highlighted by the great number of uncultured and unnamed bacterial clones that have been recovered from metagenomic surveys of microalgal blooms and colonies in freshwater environments^9^,^10^.

Symbiotic bacteria in natural water environments live in close contact with microalgae. Several symbiotic bacterial species live within microalgal cells as endosymbionts^11^,^12^,^13^, whereas others live attached to the cell walls of microalgae as episymbionts^7^,^11^,^13^,^14^,^15^. Some microalgae develop extracellular extensions to connect with other cells in order to form a colony, in or on which many bacteria may reside^16^. Such extensions are often referred to as an extracellular colony matrix, or ‘phycosphere’, and may benefit bacterial associates because they can function both as a nutrient source and residence^17^. The colony matrix composition differs between algal species, but it often contains photosynthesis-derived carbohydrates, including polysaccharides^18^. These can serve as the carbon source for symbiotic heterotrophic bacteria^17^. In return, many symbiotic bacteria are presumed to provide their microalgal counterparts with essential micronutrients for growth, such as vitamin B_12_ (cobalamin), which no algal species are known to synthesize *de novo*^19^. Micronutrients are not the only bioactive molecules that are supplied by symbiotic bacteria. Other molecules secreted from bacteria include siderophores that help algal hosts acquire iron more effectively^8^,^20^, growth promoters that stimulate algal growth^6^, and antibiotics that kill other potentially harmful microbes^21^. It is suggested that even a single bacterium can produce all of these compounds^5^.

*Botryococcus braunii* Kützing is a colony-forming green alga, taxonomically belonging to the Trebouxiophyceae, Chlorophyta^22^. *B. braunii* has been recovered from fresh and brackish water environments, such as ponds, lakes, and reservoirs worldwide. Occasionally, *B. braunii* cell densities reach very high concentrations in natural environments (1.4 × 10^6^ colonies L^−1^), resulting in the situation known as an algal bloom^23^. *B. braunii* is unique in that it accumulates a large amount of liquid hydrocarbons (up to 75% of its dry cell weight) within its colony matrix^24^. These hydrocarbons can be hydrocracked to produce the liquid transportation fuels such as gasoline, diesel, and jet fuels^25^. For this reason, *B. braunii* has attracted attention as a promising feedstock for commercial biofuel production 22,24. However, this is not currently feasible, partly because *B. braunii* grows too slowly under laboratory conditions to make biofuel production cost-effective^26^. Researchers are making an attempt to improve the biomass productivity of *B. braunii* in various ways, including nutrient and CO_2_ supply optimization and co-cultivation with bacteria^22^,^27^.

In nature, *B. braunii* harbors many epibiotic bacteria in and on its well-developed colonies^28^. Several bacterial species have been recovered from non-axenic cultures of *B. braunii*, and two of them, *Flavobacterium* sp. and *Rhizobium* sp., have been shown to have a growth-promoting effect on *B. braunii* 29,30. Both these bacteria were easily cultured and assigned to known genera that had already been taxonomically characterized. We believe that many more bacteria are associated with *B. braunii* and remain to be identified and described. Such bacteria may have some positive effects on the biomass and hydrocarbon productivity of *B. braunii*.

In this study, we describe a novel bacterium that is associated with *B. braunii*. This bacterium was isolated from a culture of *B. braunii* Ba10, a recently isolated non-axenic strain with a growth rate comparable to that of the fastest-growing *B. braunii* strains^28^. Most interestingly, this bacterium belongs to a lineage within *Alphaproteobacteria* to which all named species are distantly related. Here we demonstrate that this bacterium has a significant growth-promoting effect on its host. Our results suggest that this bacterium represents a unique lineage that is associated with phytoplankton blooms in freshwater environments worldwide.

## Results

The *B. braunii* strain Ba10^28^ isolated from a small pond in Myanmar was used for a survey of novel symbiotic bacteria. The striking feature of this strain is the presence of numerous epibiotic bacteria, and this motivated us to isolate and characterize them. Previous efforts to isolate these bacteria using the standard media for heterotrophic bacteria (trypticase soy agar, TSA) as well as the medium for *B. braunii* (AF-6,^31^ were unsuccessful^28^. Efforts using modified TSA media including vitamins [VB_6_ (1 mg L^−1^) and VB_12_ (1 mg L^−1^)], and AF-6 medium including sugars [arabinose (5 g L^−1^) and galactose (5 g L^−1^)], which are major components of the extracellular matrix of *B. braunii*^32^, were also unsuccessful. Considering that these bacteria are difficult to culture, we first determined to isolate these bacteria by co-cultivation with the host *B. braunii*.

**Figure 1:**
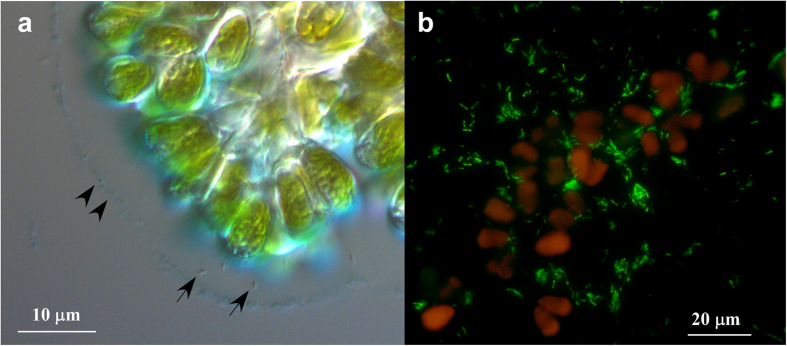
Micrographs of BOTRYCO-2 associated with *B. braunii*. (**a**) A differential interference contrast image of *B. braunii* Ba10^−/BOTRYCO−2^ showing the association of *B. braunii* and BOTRYCO-2. Alcian blue was used to stain the acidic polysaccharides. Arrowheads indicate the outer most rim of the extracellular matrix (EM). Arrows indicate BOTRYCO-2 associated with the EM. (**b**) CARD-FISH of *B. braunii* Ba10^−/BOTRYCO−2^ with the BOTRYCO-2-specific probe BAG645 (yellow-green). Algal cells are indicated in orange because of chlorophyll autofluorescence.

**Figure 2:**
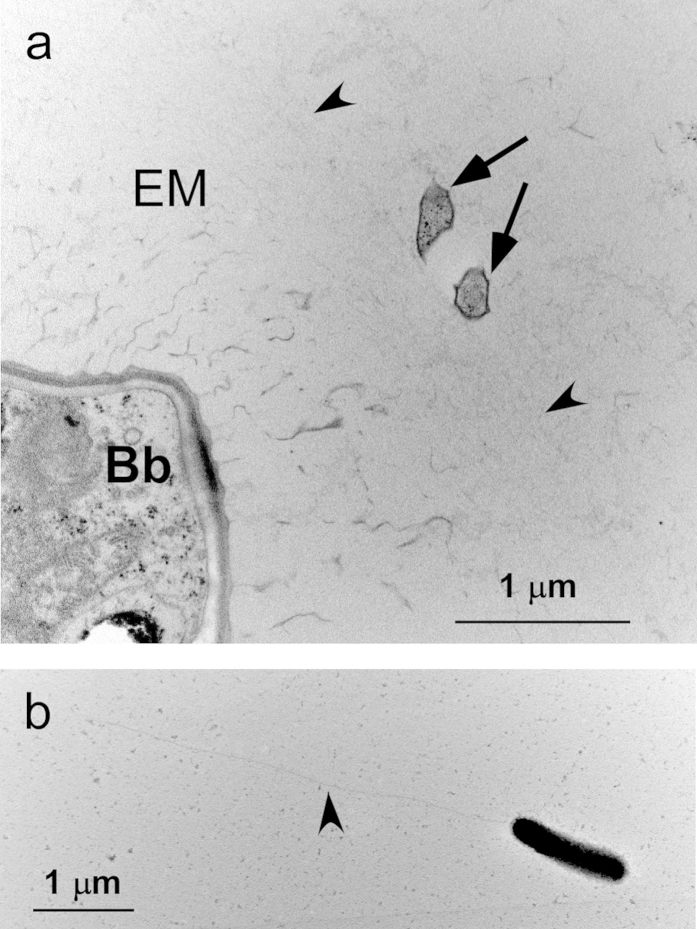
TEM images. (**a**) TEM image of an ultrathin section of *B. braunii* Ba10^−/BOTRYCO−2^. Arrows, BOTRYCO-2; Bb, a cell of *B. braunii*; EM, the extracellular matrix of *B. braunii*. Arrowheads indicate the outermost rim of the EM. Note that bacteria do not appear rod-shaped in the ultrathin section. (**b**) a whole mount positive-stained TEM image of BOTRYCO-2. Arrowhead indicates a flagellum.

By inoculating the algae-free bacterial suspension prepared from *B. braunii* Ba10 into the axenic culture of Ba10 (hereafter denoted as Ba10^−^), two cultures of Ba10^−^, each of which comprises a single but different bacterial morphotype, were successfully established. Here we characterize one of the two bacteria, designated BOTRYCO-2. The presence of a single morphological species of bacteria in the co-culture (Ba10^−^ plus BOTRYCO-2) was confirmed by TEM analysis (Supplementary Fig. S1). The presence of a single 16S rDNA genotype in the co-culture was confirmed by the results of 16S rDNA sequencing and clone library analyses. Ba10^−^ inoculated with this bacterial strain was denoted Ba10^−/BOTRYCO−2^. Light microscopic observation (Fig. 1a) and the CARD-FISH (Fig. 1b) experiment of Ba10^−/BOTRYCO−2^ showed that the bacterium is either associated with the *B. braunii* extracellular matrix or is free living in the liquid medium. In Fig. 1a, it was unclear whether BOTRYCO-2 was present inside or on the matrix. TEM photo of an ultrathin section of the algal colony of Ba10^−/BOTRYCO−2^ showed that the colony-associated BOTRYCO-2 was attached to the outer surface of the extracellular matrix of *B. braunii* (Fig. 2a). The intrusion of the bacterium into the matrix was not observed in multiple TEM observations. Morphologically, BOTRYCO-2 is a rod-shaped bacterium that is normally 1.5–2 μm in length; however, bacterial cells that were double this length appeared synchronously during some growth periods (Supplementary Fig. S1). The positive-stained TEM image (Fig. 2b) indicated the presence of a single flagellum (approximately 4 μm) in the posterior region of the bacterial cell.

**Figure 3:**
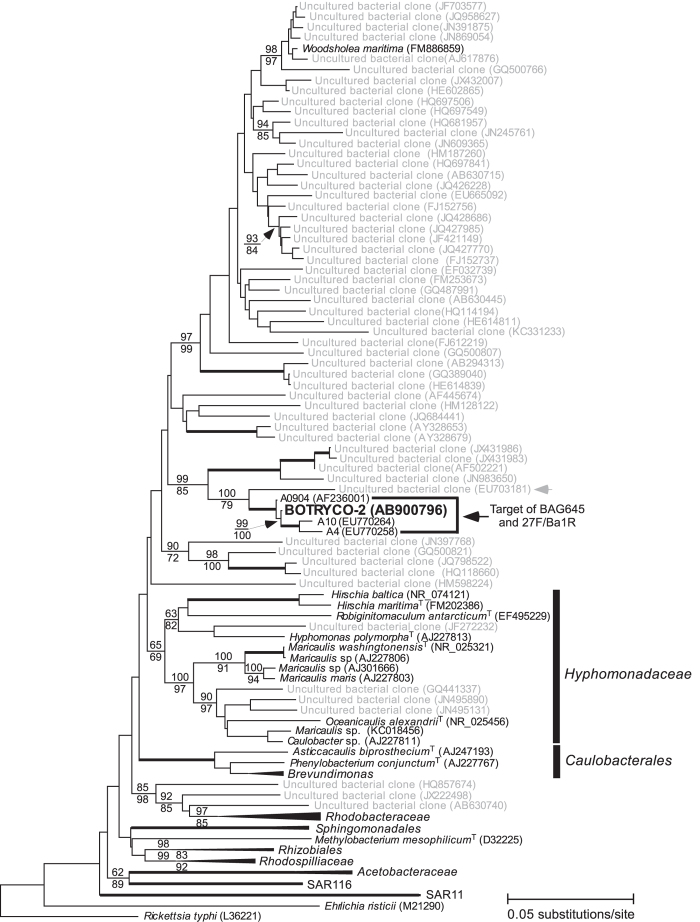
A 16S rDNA NJ tree of *Alphaproteobacteria* focusing on the phylogenetic location of BOTRYCO-2. Numbers in parentheses indicate the GenBank accession numbers. The tree was rooted with *Rickettsia typhi* (L36221). ^T^ indicates the type strain of the bacterial species. Bootstrap values (>60%) on the basis of NJ and ML are shown above and below the branches, respectively, whereas the values for branches that are not consistent between the NJ and ML trees are not shown. Branches obtaining 100% values for both NJ and ML are indicated in bold. Well-defined clades that are distantly related to BOTRYCO-2 are compressed. A full version of the NJ tree including all OTUs is provided in Supplementary Fig. S10. The target of the BOTRYCO-2-specific probe and primer is indicated. Note that the corresponding region within EU703181 (indicated by the gray arrow) is 1 bp different from the probe sequence, so that EU703181 may be detected by FISH and PCR under less stringent conditions.

**Figure 4:**
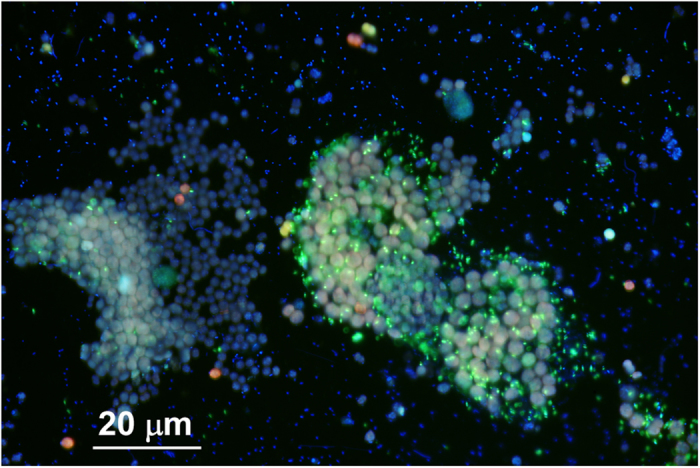
The merged fluorescent image (DAPI staining and CARD-FISH) of a *M. aeruginosa* bloom sample. *M. aeruginosa* cells appear pink or pinkish blue on account of chlorophyll autofluorescence. Blue signals are from bacteria negative for the BAG645 probe. Spotted positive signals (yellow-green) of the BAG645 probe are observed mostly from bacteria associated with the colony and cells of *M. aeruginosa*, not from free-living bacteria. Note that two morphological types of *M. aeruginosa*, differing in cell size, are found in the sample and that the FISH signals are more intense in the right-hand colony.

The BLAST search of BOTRYCO-2 16S rDNA indicated that this bacterium belongs to the class *Alphaproteobacteria*, but no named species with sequence identity >90% to that of BOTRYCO-2 were found. The most closely related named species was *Maricaulis washingtonensis* (pairwise sequence identity, 89.8%), a marine bacterium in the family *Hyphomonadaceae*^33^. Most sequences showing >90% DNA identity to that of BOTRYCO-2 were obtained from uncultured environmental clones from aquatic freshwater environments, including algal colonies. Phylogenetic analyses of allied *Alphaproteobacteria* located BOTRYCO-2 within a clade comprising two uncharacterized bacterial isolates A4 and A10 (GenBank accession numbers EU770258 and EU770264, respectively), one environmental clone from a freshwater environment (EU703181), and one sequence from an unknown source (AF236001) with high statistical support (Fig. 3). These four 16S rDNA sequences showed 99% identity to the sequence of BOTRYCO-2, whereas all other sequences showed <94% identity. In the global alphaproteobacterial phylogeny, this clade and several other lineages that include exclusively environmental clones (with the one exception of *Woodsholea maritima*) form a sister lineage to the *Hyphomonadaceae* clade, although the bootstrap statistical supports were weak (<50%, both in NJ and ML). According to the study that detected the A4 and A10 sequences, they both originated from a colony of the cyanobacterium *Microcystis aeruginosa*^34^. This organism is one of the main constituents of cyanobacterial water blooms that occur in eutrophic freshwater environments worldwide^35^. To investigate a possible association of BOTRYCO-2-related bacteria with *M. aeruginosa*, CARD-FISH analysis with the probe specific for BOTRYCO-2 related bacteria (BAG645, Supplementary Fig. S2) was performed using a water bloom sample containing *M. aeruginosa*. The results indicated that the bloom sample was positive for BOTRYCO-2, with the FISH signal found mostly in bacteria associated with colonies of *M. aeruginosa* rather than in free-living bacteria (Fig. 4). Of note, the FISH signal was mostly associated with one of two types of colony of *M. aeruginosa* present in the sample. A PCR survey using *M. aeruginosa*-containing environmental water bloom samples from Japan, other Asian regions and Kenya indicated that 35 of the 39 bloom samples were positive for BOTRYCO-2-related sequences; a positive signal was also detected in an environmental sample of *B. braunii* (Supplementary Table S1). Direct sequencing experiments of the PCR amplicons indicated a diversity of BOTRYCO-2-related sequences, including at least four different genotypes (inferred from the redundancy of SNPs) (Supplementary Table S1). One of the recovered genotypes was the same as the genotype of BOTRYCO-2. More than two genotypes were frequently recovered from a single bloom.

Although our initial efforts to obtain an axenic culture of BOTRYCO-2 were unsuccessful as described above, we finally succeeded using the low nutrient medium employed to cultivate the related alphaproteobacterial species A4 and A10^34^. The colour of the colonies on agar plates is burgundy red, which is consistent with that of the bacterial cell filtrates from *B. braunii* co-cultures (Supplementary Fig. S3). The cell morphology of BOTRYCO-2 observed in the axenic culture matched perfectly with that seen in co-culture. However, it should be noted that the number of the recovered colonies of BOTRYCO-2 was approximately 10. This is considerably smaller than the number of bacterial cells in the inoculum (>10^5^ cells). In addition, the axenic growth of BOTRYCO-2 was unstable, as it failed to grow in the axenic plate medium following 3–4 subcultures of the initial colony.

**Figure 5:**
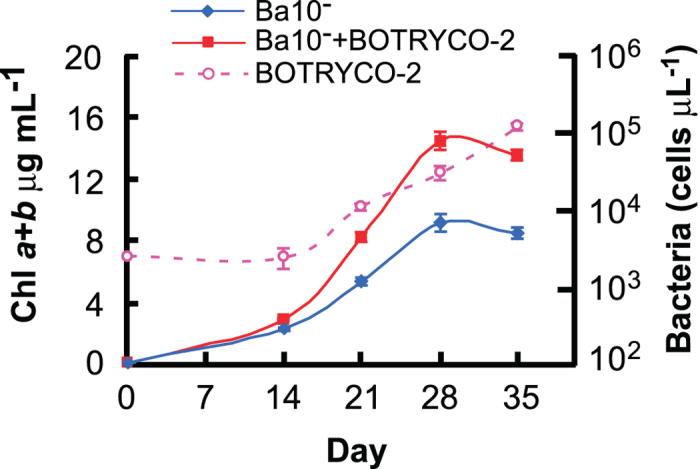
Effect of BOTRYCO-2 on the growth of *B. braunii* Ba10^−^. The first y-axis shows chlorophyll a + b concentration of Ba10− with and without inoculation of BOTRYCO-2 and the second y-axis shows cell concentrations of BOTRYCO-2 in the culture of Ba10^−^ with BOTRYCO-2. Bars indicate the standard error of four biological replicates.

**Figure 6:**
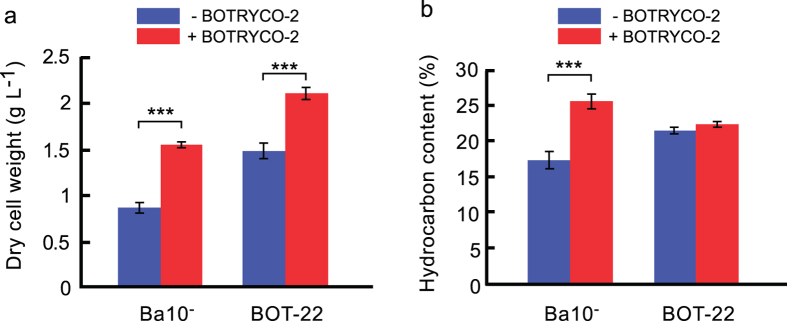
Effect of BOTRYCO-2 on the biomass and hydrocarbon productivities of *B. braunii*. Final biomass concentrations (**a**) and hydrocarbon contents (**b**) of *B. braunii* with or without BOTRYCO-2 on the basis of the growth experiments depicted in Fig. 5 and Supplementary Fig. S7 (at days 35 and 49 for Ba10^−^ and BOT-22, respectively). The y-axes indicate dry cell weight (dcw) per culture volume and hydrocarbon content (w dcw^−1^) for **a** and **b**, respectively. Bars indicate the standard error of four and five biological replicates for Ba10^−^ and BOT-22, respectively. The final biomass concentrations of Ba10^−^ and BOT-22 with BOTRYCO-2 were significantly higher than those without BOTRYCO-2 (homoscedastic one-tailed t-test, *P* = 0.00003 and *P* = 0.00007, respectively). The hydrocarbon content of Ba10^−^ with BOTRYCO-2 was significantly higher than that without BOTRYCO-2 (homoscedastic one-tailed t-test, *P* = 0.00007), whereas BOT-22 showed no significant difference between cultures with and without BOTRYCO-2 (homoscedastic two-tailed t-test, *P* = 0.107). *** indicates significant differences between with and without BOTRYCO-2 (*P* < 0.001).

To investigate the possible influence of BOTRYCO-2 on the growth of *B. braunii* Ba10^−^, comparative growth experiments were performed. The *de novo* BOTRYCO-2-inoculated cultures of Ba10^−^ showed elevated growth compared with Ba10^−^ without BOTRYCO-2 (approximately 1.8-fold increase in biomass) (Figs. 5 and 6a), indicating the positive effect of BOTRYCO-2 on the growth of Ba10^−^. BOTRYCO-2 cell concentration in the culture also increased concomitantly with algal growth, but with a lag of 14 days (Fig. 5). The presence of BOTRYCO-2 had no effects on pH curves during the culture period (Supplementary Fig. S4). Growth experiments of Ba10^−/BOTRYCO−2^ using an antibiotic-treated culture as a control also indicated a positive effect on the growth of *B. braunii* in the presence of BOTRYCO-2 (Supplementary Figs. S5). An inoculation experiment using the axenic strain *B. braunii* BOT-22, a strain closely related to Ba10, both of which belonging to ‘race B’ (Supplementary Fig. S6), showed a different effect. The growth rate of BOT-22 was similar with and without BOTRYCO-2 (Supplementary Fig. S7), whereas the final biomass concentration of BOT-22 with BOTRYCO-2 was approximately 1.4 times higher than that without BOTRYCO-2 (Fig. 6a). The hydrocarbon content of Ba10^−^ with BOTRYCO-2 was approximately 1.5 times higher than that without, whereas the hydrocarbon content of BOT-22 was similar regardless of the presence of BOTRYCO-2 (Fig. 6b).

## Discussion

Here we have described a novel alphaproteobacterium, designated BOTRYCO-2, isolated from a non-axenic field isolate of *B. braunii*. 16S rDNA phylogenetic analyses suggested a unique phylogenetic position for this bacterium in an enigmatic lineage where virtually all related sequences are from uncultured environmental clones. The analyses also suggested a low phylogenetic affinity of BOTRYCO-2 to the *Hyphomonadaceae* and *Caulobacterales* families. These are both characterized by the possession of stalk-like structures called prostheca and an asymmetric cell division system that gives rise to a motile daughter cell from the non-motile mother cell^36^. These features are also found in *W. maritima*^37^, a single species in a weakly supported clade encompassing BOTRYCO-2, and a battery of additional environmental clones neighbouring the *Hyphomonadaceae* clade. However, these morphological features have not been observed in BOTRYCO-2, at least under our culture conditions. This suggests that BOTRYCO-2 can be taxonomically delineated from the *Caulobacterales* and *Hyphomonadaceae* in terms of morphology. The presence of several poorly understood lineages neighbouring the *Hyphomonadaceae* clade is evident from the phylogeny, and most bacterial strains in these lineages have yet to be isolated and characterized. Given that BOTRYCO-2 and its close relatives appear abundant and widespread in eutrophic freshwater environments during algal blooms, it is likely that BOTRYCO-2 and its relatives are among the diverse bacteria that preferentially feed on algal colonies that produce specific carbohydrates and micronutrients. Our experimental protocol employing the dilution-based bacterial inoculation to an axenic algal culture is simple but highly promising for further exploration for such bacterial associates, especially for bacterial species that cannot be cultured without their algal hosts. Because BOTRYCO-2 shows a low sequence identity to known bacterial species (<90%), we conclude that this strain, and possibly other four sequences (EU703181, EU770258, EU770264, and AF236001) represent novel species. On the basis of its close association with algae and rod-shaped form, we tentatively propose the name ‘*Candidatus* Phycosocius bacilliformis’ for BOTRYCO-2 [from the Latin *Phyco* (Algae), *socius* (associated), and *bacilliformis* (rod-shaped)]. If a stable axenic culture condition can be found, a full taxonomic description of this species is feasible.

Growth experiments suggested that BOTRYCO-2 confers higher biomass productivity to *B. braunii*. It is known that pH influences the available CO_2_ forms [CO_2_ (aq), bicarbonate and carbonate] and their amounts in liquid media^38^. The pH curves during the culture period did not differ regardless of the presence of BOTRYCO-2, suggesting that growth enhancement of the alga was not an indirect effect due to differing CO_2_ availability. Given that BOTRYCO-2 cannot grow without *B. braunii* in the phototrophic medium and that heterotrophic bacteria cannot grow without external carbon sources, we hypothesize that BOTRYCO-2 feeds on components of the extracellular matrix of *B. braunii*. These are likely to be polysaccharides, long-chain hydrocarbons (botryococcenes), or their derivatives (algaenans), all of which have been shown to reside within the *B. braunii* colony matrix^32^. BOTRYCO-2 is found both unattached and attached to its host, suggesting that BOTRYCO-2 may utilize both diffused and cell-associated carbons. In return, *B. braunii* presumably obtains some as yet unknown elements from BOTRYCO-2, which results in the observed higher biomass production. One possible growth-promoting group of elements that is known to be essential for the growth of many microalgae is vitamins^19^. In this context, the fact that BOTRYCO-2 showed different impacts on the growth of the cobalamin-autotrophic strain of *B. braunii*, BOT-22^39^, is noteworthy. It is possible that Ba10 requires cobalamin (supplied by BOTRYCO-2) for growth and that its growth is thus promoted in the presence of BOTRYCO-2. However, available data suggests that this is unlikely because cobalamin concentration in the growth experiment media was 738 pM (1 mg L^−1^), which is an order of magnitude higher than the maximum known concentration required for achieving the highest cell yields in cobalamin-auxotrophic microalgae in batch cultures^40^. Similarly, thiamin and biotin concentrations in the medium are 29.6 nM and 8.19 nM, respectively, an amount at which even the biomass of algae requiring the largest amount of each vitamin was shown to saturate^40^. It is possible that *B. braunii* requires unexpectedly high concentration of vitamins. To further investigate this issue, the vitamin dependencies of Ba10^−^ should be determined.

Other possibilities include enhanced nutrient availability via bacterial remineralization^41^,^42^, siderophore production facilitating the effective uptake of ferric iron^8^, oxygen scavenging by the bacterium to reduce oxidative stress^43^,^44^ or nitrogen supplied by the bacterium through nitrogen fixation^45^, all of which have been shown to stimulate the growth of algae. Omics-based approaches would clarify which mechanisms are responsible for the enhanced growth of *B. braunii*. In any event, the association between BOTRYCO-2 and *B. braunii* is likely mutualistic.

Given that BOTRYCO-2 has the ability to promote the growth of *B. braunii*, it may play a role in the natural occurrence of *B. braunii* water blooms. Unfortunately, *B. braunii* water blooms are uncommon, making it difficult to investigate this possibility. In this context, it is interesting that BOTRYCO-2 or its relatives have a high affinity for the phytoplankton *M. aeruginosa*, which is commonly associated with blooms in freshwater environments worldwide. Phylogenetically, *B. braunii* and *M. aeruginosa* are unrelated. However, they both share the ability to build polysaccharide-rich colonies^22^,^46^. Either both *B. braunii* and *M. aeruginosa* colonies produce similar carbon sources favored by BOTRYCO-2 and related bacteria or these bacteria have preferences for different carbon sources secreted by the two algae. Interestingly, the FISH experiment revealed a possible preference of BOTRYCO-2-related bacteria for a specific colony type of *M. aeruginosa*. This can be explained by the preference of BOTRYCO-2-related bacteria for specific carbon sources because polysaccharide contents differ among different *M. aeruginosa* strains^46^. Given that there was no positive effect of the BOTRYCO-2-related strains A4 and A10 on the growth of *M. aeruginosa*^34^, it is possible that BOTRYCO-2 relatives are ecologically diverse, encompassing both mutualists and endocommensals. *In vitro* culture experiments and *in situ* field surveys dealing with the temporal succession of the biomass of BOTRYCO-2-related bacteria in association with *B. braunii* and *M. aeruginosa* population dynamics should clarify how these bacteria are involved in algal bloom dynamics. In any event, given the common co-occurrence of BOTRYCO-2-related bacteria and *M. aeruginosa*, the bacteria may play a role in freshwater carbon cycling during a bloom^47^ because they assimilate photosynthates present in live algal colonies. It is also important to investigate how frequent BOTRYCO-2 is associated with *B. braunii* in nature. Such studies are essential to verify whether the observed relationship between *B. braunii* and BOTRYCO-2 represents a natural long-standing symbiosis or an occasional association that is highlighted in our two-member culture experiments.

*B. braunii* is an attractive candidate feedstock for commercial algal biofuel production because, unlike most other microalgae, it can produce high-quality hydrocarbons^22^. However, from a biological point of view, two major challenges remain to be overcome before commercial biofuel production becomes feasible. First, the oil productivity of *B. braunii* is lower than those of other microalgal species^48^. Second, large-scale cultivation of *B. braunii* has not been successful^48^. Because BOTRYCO-2 does not markedly enhance the hydrocarbon productivity of *B. braunii*, the first issue remains unresolved. On the other hand, the second challenge, ‘scale-up problem’, may be resolved in the presence of symbiotic bacteria. Previous efforts aiming at the large-scale cultivation of *B. braunii* have focused on avoiding contamination^49^. Because *B. braunii* blooms occur in nature in the co-existence of numerous microbes, we speculate that higher cell concentrations of *B. braunii* in artificial ponds could be achieved in the presence of good symbiotic microbial associates such as BOTRYCO-2.

## Methods

### Single cell isolation of associated bacteria

A late logarithmic to stationary phase culture of *B. braunii* Ba10^28^ was used for bacterial isolation. After confirming the presence of bacteria under a microscope, the culture was vortexed for 1 min and was gently filtered in a sterile chamber using a GF/C filter (pore size: 1.2 μm; Whatman, Buckinghamshire, UK). Because *B. braunii* cells are >5 μm in length, only bacterial cells were recovered in the filtrate. The bacterial cell suspension was filtered using a Nuclepore polycarbonate filter (pore size: 0.2 μm; Whatman). The filter embedded with bacteria was dried, placed on a glass slide and covered with 10 μL of antifading reagent [Citifluor (Citifluor, UK): Vectashield (Vector Laboratories, Burlingame, CA) = 4:1] including DAPI (1 μg mL^−1^). The cell number was counted under a fluorescence microscope to estimate bacterial concentration in the filtrate. On the basis of the estimated cell concentration, the filtrate was diluted and inoculated into an axenic culture of Ba10 (Ba10^−^) in a 1.5-mL microtube in such a way that, statistically, one bacterial cell was present in each tube. The microtubes were incubated for >3 weeks, when numerous bacterial cells were visible around the *B. braunii* colonies under the microscope. The co-culture of the bacterium and *B. braunii* Ba10^−^ was maintained for >4 months through 2–3 times of serial transfer into new media before the growth experiments. The protocol for establishing the axenic culture of *B. braunii* Ba10 (Ba10^−^) (Supplementary Fig. S8) is described in Supplementary Method.

### 16S rDNA analysis

16S rDNA of BOTRYCO-2 was PCR amplified using the bacterial universal primer pair 27F/1492R^50^. The determined 16S rDNA sequence of the bacterium has been deposited in the DNA Databank of Japan (DDBJ) under the accession number AB900796. Phylogenetic analyses were performed on the basis of neighbour-joining (NJ) and maximum-likelihood (ML) methods using MEGA version 5.2^51^ and RAxML^52^, respectively. The detailed protocols for DNA extraction, PCR, and sequencing reactions are described in Supplementary Method.

### Catalyzed reporter deposition fluorescence *in situ* hybridization.

The CARD-FISH probe (BAG645, 5′- ACTTTggTCCAgATACCC -3′, corresponding to the *Escherichia coli* positions 645–662) specific to the recovered bacterial 16S rDNA sequence and its closest relatives (Fig. 3), was designed manually on the basis of the alignment described above with the help of the published relative fluorescence intensity information^53^. The specificity of the probe was checked using the Probe Match tool of the Ribosomal Database Project (http://rdp.cme.msu.edu/), resulting in at least two mismatches to 16S rDNA sequences of non-target bacteria. A culture in the mid-to-late exponential growth phase of *B. braunii* was used for CARD-FISH analyses. The basic protocol of CARD-FISH was as described previously^54^. Formamide concentrations for the positive (EUB338 I–III) and negative (NON338) control probes were based on the published protocol^55^, whereas the optimal formamide concentration for BAG645 was determined as 20% (v/v). All samples were confirmed negative in CARD-FISH experiments with NON338.

### Transmission electron microscopy (TEM)

To visualize bacterial cells and flagella, a positive TEM staining was performed by adding EM grade glutaraldehyde [10% (v/v) of 25%] to cell suspension (final concentration, 2.5%). Fixation was performed at 4 °C for 24 h. Cells were fixed in osmium tetroxide vapor on formvar-coated copper grids at room temperature for 20 min. After rinsing with distilled water, the grids were triple stained with drops of 2% uranyl acetate, 2% tannic acid and Sato’s lead solution^56^. Ultrathin sections of *B. braunii* colonies with BOTRYCO-2 were prepared using a cryofixation-based technique^57^. Samples were examined using a transmission electric microscope, H-7650 (Hitachi, Tokyo, Japan).

### Growth experiments

Instead of measuring optical density (OD), the growth of *B. braunii* was assessed on the basis of chlorophyll *a* + *b* concentration to avoid the overestimation of OD due to a possible bacterial contribution. Chlorophyll concentration has been shown to be useful in estimating the biomass of *B. braunii* during early to mid growth period^58^. At each measuring time point, *B. braunii* cells were collected by GF/C filtration of 0.5–2 mL culture, followed by chlorophyll extraction using methanol. ODs at 665 and 650 nm were measured using a UV spectrophotometer (UV-1800; Shimadzu, Kyoto, Japan), and the chlorophyll *a* + *b* concentration was calculated using the canonical equation^59^.

The bacterial suspension used for *de novo* inoculation experiments was re-isolated from Ba10^−/BOTRYCO−2^ in the same manner as in the initial single bacterial cell isolation. The final concentration of the bacterium in inoculation experiments was adjusted to the ratio of the chlorophyll *a* + *b* concentration and bacterial cell counts in the mid-to-late logarithmic growth phase of Ba10^−/BOTRYCO−2^, assuming that bacterial concentrations relative to the host is optimum at this time point. Preliminary experiments indicated that this ratio is approximately 10^6^–10^7^ bacterial cells [chl *a* + *b*] μg^−1^. An axenic culture of *B. braunii* BOT-22^60^ was also used in the *de novo* inoculation experiments. All cultures were grown in 60 mL AF-6 medium in 100-mL test tubes at 25 °C under the continuous light of 50 μmol photon m^−2^ s^−1^ with approximately 15 mL min^−1^ of 1% CO_2_ aeration. The 1% CO_2_ aeration was reduced to approximately 2.5 mL min^−1^ in the late growth period to avoid the generation of excess bubbles in the test tube. A late logarithmic to early stationary growth phase culture (1.0–1.5 g [dcw] L^−1^) was used as the inoculum. All culture experiments were performed with three to five biological replicates. Ampicillin (final concentration, 50 μg mL^−1^) was used to kill the symbiotic bacterium in the control experiment. Ampicillin at this concentration does not affect the growth of *B. braunii* (Supplementary Fig. S9). The presence of the single bacterial morphotype and genotype throughout the culture period was confirmed by microscopic observation and by direct sequencing of 27F/1492R PCR products. Bacterial cell (BOTRYCO-2) concentrations at each sampling point were estimated using DAPI staining-based cell counting, as described above. No DAPI signal was observed at any sampling point in the Ba10^−^ culture without inoculating BOTRYCO-2. Dry cell weight of *B. braunii* was measured after GF/C filtration. Hydrocarbon was extracted and measured following the published protocol^28^.

## Additional Information

**How to cite this article**: Tanabe, Y. *et al*. A novel alphaproteobacterial ectosymbiont promotes the growth of the hydrocarbon-rich green alga *Botryococcus braunii*. *Sci. Rep.* **5**, 10467; doi: 10.1038/srep10467 (2015).

## Supplementary Material

Supplementary Information
