# Supplementary material for: A novel alphaproteobacterial ectosymbiont promotes the growth of the hydrocarbon-rich green alga Botryococcus braunii
Source: Sci Rep. 2015 Jul 1;5:10467. doi: 10.1038/srep10467 (PMC4486929; doi:10.1038/srep10467)
Supplement: Supplementary Information [file srep10467-s1.pdf]

Supplementary information

A novel alphaproteobacterial ectosymbiont promotes the growth of the hydrocarbon-rich green alga *Botryococcus braunii*

Yuuhiko Tanabe*[1], Yusuke Okazaki[2], Masaki Yoshida[1], Hiroshi Matsuura[1], Atsushi Kai[1], Takashi Shiratori[3], Ken-ichiro Ishida[1], Shin-ichi Nakano[2], Makoto M. Watanabe[1]

[1] Faculty of Life & Environmental Sciences, University of Tsukuba

[2] Center for Ecological Research, Kyoto University

[3] Graduate School of Life & Environmental Sciences, University of Tsukuba

*Corresponding author: tanabe.yuuhiko.fn@u.tsukuba.ac.jp

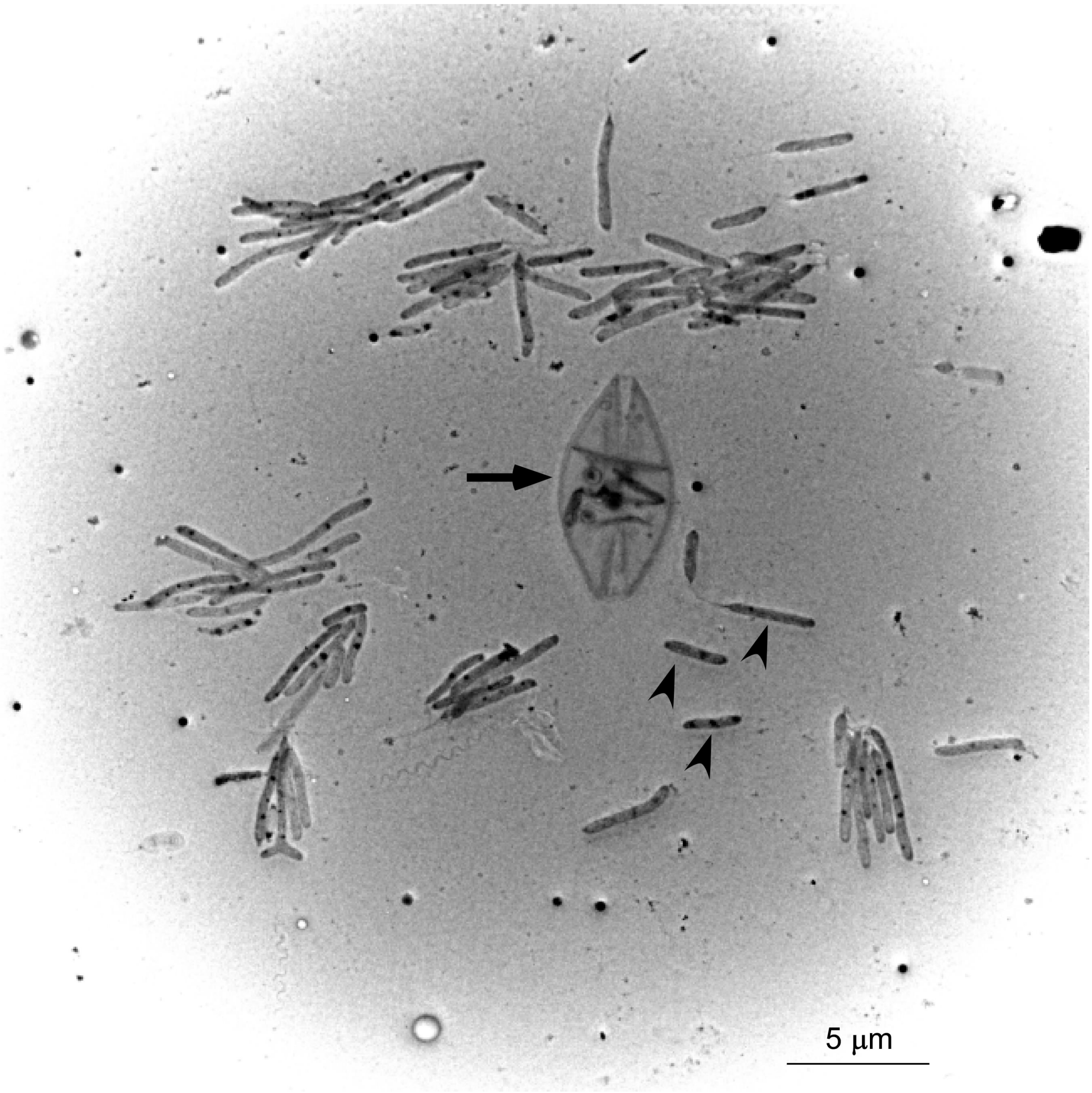

Figure S1

TEM image of *B. braunii* Ba10$^{-/BOTRYCO-2}$ culture on a whole-mount preparation showing the morphological uniformity of bacterial cells (arrowheads). Note that more than half of the putative BOTRYCO-2 cells are double the size of the normal cells (Fig. 2b). This is either due to imminent cell division, or to cell dimorphism in response to environmental changes, or both. The arrow indicates a ‘shell’ of a cell of *B. braunii* (32). The sample for TEM was prepared as described in Methods except that 2 % tannic acid was not used for staining.

a

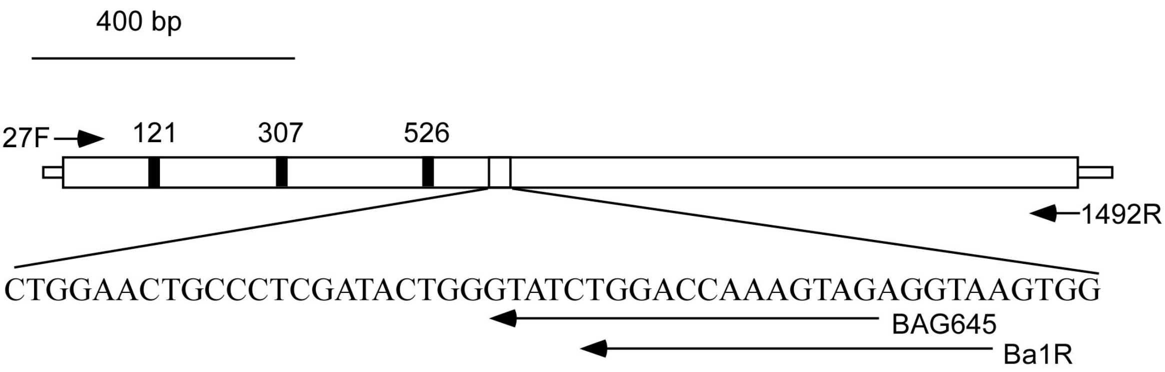

b

```
BOTRYCO_2        AGCGAACGCTGGCGGCAGGCTTAACACATGCAAGTCGAACGCCATCTTCGGATGGAGTGG
091023_Mizube    AGCGAACGCTGGCGGCAGGCTTAACACATGCAAGTCGAACGCCATCTTCGGATGGAGTGG
100823_kenya     AGCGAACGCTGGCGGCAGGCTTAACACATGCAAGTCGAACGCCATCTTCGGATGGAGTGG
A10              ------GGGGAAGGGGGGGCTTTAAA-ATGCAAGTCGAACGCCATCTTCGGATGGAGTGG
A4               --------TAAATGCGCGGCT---ACCGTGCGAGTCGA-CGCCATCTTCGGATGGAGTGG
                              *   ****       *** ****** *********************

BOTRYCO_2        CAGACGGGTGAGTAACGCGTGGGAACGTACCCTTTGGTTCGGAATATTCTCGGGAAACTG
091023_Mizube    CAGACGGGTGAGTAACGCGTGGGAACGTACCCTTTGGTTCGGAATATTCTCGGGAAACTG
100823_kenya     CAGACGGGTGAGTAACGCGTGGGAACGTACCCTTTGGTTCGGAATATTCTCGGGAAACTG
A10              CAGACGGGTGAGTAACGCGTGGGAACGTACCCTTTGGTTCGGAATATTCTCGGGAAACTG
A4               CAGACGGGTGAGTAACGCGTGGGAACGTACCCTTTGGTTCGGAATATTCTCGGGAAACTG
                 ************************************************************

                121
BOTRYCO_2        GGAGCAATACCGGATAAGCCCTACGGGGGAAAGATTTATCGCCAAAGGAGCGGCCCGCGT
091023_Mizube    MGAGCAATACCGGATAAGCCCTACGGGGGAAAGATTTATCGCCAAAGGAGCGGCCCGCGT
100823_kenya     AGAGCAATACCGGATAAGCCCTACGGGGGAAAGATTTATCGCCAAAGGAGCGGCCCGCGT
A10              AGAGCAATACCGGATAAGCCCTACGGGGGAAAGATTTATCGCCAAAGGAGCGGCCCGCGT
A4               AGAGCAATACCGGATAAGCCCTACGGGGGAAAGATTTATCGCCAAAGGAGCGGCCCGCGT
                  ***********************************************************

BOTRYCO_2        CAGATTAGGTAGTTGGTGGGGTAAAGGCCTACCAAGCCTACGATCTGTAGCTGGTCTGAG
091023_Mizube    CAGATTAGGTAGTTGGTGGGGTAAAGGCCTACCAAGCCTACGATCTGTAGCTGGTCTGAG
100823_kenya     CAGATTAGGTAGTTGGTGGGGTAAAGGCCTACCAAGCCTACGATCTGTAGCTGGTCTGAG
A10              CAGATTAGGTAGTTGGTGGGGTAAAGGCCTACCAAGCCTACGATCTGTAGCTGGTCTGAG
A4               CAGATTAGGTAGTTGGTGGGGTAAAGGCCTACCAAGCCTACGATCTGTAGCTGGTCTGAG
                 ************************************************************
```

```
BOTRYCO_2        AGGATGATCAGCCACACTGGGACTGAGACACGGCCCAGACTCCTACGGGAGGCAGCAGTG
091023_Mizube    AGGATGATCAGCCACACTGGGACTGAGACACGGCCCAGACTCCTACGGGAGGCAGCAGTG
100823_kenya     AGGATGATCAGCCACACTGGGACTGAGACACGGCCCAGACTCCTACGGGAGGCAGCAGTG
A10              AGGATGATCAGCCACACTGGGACTGAGACACGGCCCAGACTCCTACGGGAGGCAGCAGTG
A4               AGGATGATCAGCCACACTGGGACTGAGACACGGCCCAGACTCCTACGGGAGGCAGCAGTG
                 ************************************************************

                       307
BOTRYCO_2        GGGAATCTTGGACAATGGGCGCAAGCCTGATCCAGCCATGCCGCGTGGATGATGAAGGCC
091023_Mizube    GGGAATCTTGGACAATGGGCGCAAGCCTGATCCAGCCATGCCGCGTGGATGATGAAGGCC
100823_kenya     GGGAATCTTGGACAATGGGCGCAAGCCTGATCCAGCCATGCCGCGTGGATGATGAAGGCC
A10              GGGAATCTTGGACAATGGGCGCAAGCCTGATCCAGCCATGCCGCGTGGATGATGAAGGCC
A4               GGGAATATTGGACAATGGGCGCAAGCCTGATCCAGCCATGCCGCGTGGATGATGAAGGCC
                 ****** *****************************************************

BOTRYCO_2        TTAGGGTTGTAAAATCCTTTCAACGGGGACGATAATGACGGTACCCGTAGAAGAAGCCCC
091023_Mizube    TTAGGGTTGTAAAATCCTTTCAACGGGGACGATAATGACGGTACCCGTAGAAGAAGCCCC
100823_kenya     TTAGGGTTGTAAAATCCTTTCAACGGGGACGATAATGACGGTACCCGTAGAAGAAGCCCC
A10              TTAGGGTTGTAAAATCCTTTCAACGGGGACGATAATGACGGTACCCGTAGAAGAAGCCCC
A4               TTAGGGTTGTAAAATCCTTTCAACGGGGACGATAATGACGGTACCCGTAGAAGAAGCCCC
                 ************************************************************

BOTRYCO_2        GGCTAACTTCGTGCCAGCAGCCGCGGTAATACGAAGGGGGCTAGCGTTGCTCGGAATTAC
091023_Mizube    GGCTAACTTCGTGCCAGCAGCCGCGGTAATACGAAGGGGGCTAGCGTTGCTCGGAATTAC
100823_kenya     GGCTAACTTCGTGCCAGCAGCCGCGGTAATACGAAGGGGGCTAGCGTTGCTCGGAATTAC
A10              GGCTAACTTCGTGCCAGCAGCCGCGGTAATACGAAGGGGGCTAGCGTTGCTCGGAATTAC
A4               GGCTAACTTCGTGCCAGCAGCCGCGGTAATACGAAGGGGGCTAGCGTTGCTCGGAATTAC
                 ************************************************************

                                                             526
BOTRYCO_2        TGGGCGTAAAGCGCACGTAGGCGGATATTTAAGTCGGAGGTGAAAGCCCAGGGCTCAACC
091023_Mizube    TGGGCGTAAAGCGCACGTAGGCGGATATTTAAGTCGGAGGTGAAAKCCCAGGGCTCAACC
100823_kenya     TGGGCGTAAAGCGCACGTAGGCGGATATTTAAGTCGGAGGTGAAATCCCAGGGCTCAACC
A10              TGGGCGTAAAGCGCACGTAGGCGGATATTTAAGTCGGAGGTGAAATCCCAGGGCTCAACC
A4               TGGGCGTAAAGCGCACGTAGGCGGATATTTAAGTCGGAGGTGAAAGCCCAGGGCTCAACC
                 ********************************************* **************
```

```
BOTRYCO_2         CTGGAACTGCCCTCGATACTGGGT
091023_Mizube     CTGGAACTGCCCTCGATACTGGGT
100823_kenya      CTGGAACTGCCCTCGATACTGGGT
A10               CTGGAACTGCCCTCGATACTGGGT
A4                CTGGAACTGCCCTCGATACTGGGT
                  ************************
```

Figure S2

PCR detection of polymorphic sites in the partial 16S rDNA segments of BOTRYCO-2 relatives. For the PCR survey of environmental water samples for BOTRYCO-2-related bacteria, genomic DNAs of environmental samples were prepared using a FastDNA SPIN kit (MP Biomedicals, Tokyo, Japan). A specific primer Ba1R (5′- CCTCTACTTTggTCCAgAT -3′) was designed on the basis of the alignment obtained in the course of the phylogenetic reconstruction. The primer pair 27F/Ba1R was used to amplify the 5′ region of 16S rDNA of target bacteria (the expected amplicon length is 603 bp). The PCR condition for this amplification was the same as that for the 27F/1492R amplification except that the elongation time was set to 1 min. **a** The location of specific primer Ba1R and the FISH probe within 16S rDNA. Three polymorphic sites between BOTRYCO-2-related sequences recovered by the 27F/Ba1R PCR detection are boxed (see below). **b** The alignment of the 27F/Ba1R amplicons including five selected sequences. * indicates the same nucleotide. Three polymorphic sites within the segment are indicated in red (sites are numbered from the first site of the amplicons without including 27F primer). The polymorphic sites near the 5′ end of the 16S rDNA of A4 and A10 are ambiguous (shaded); no polymorphism in this region was detected in the PCR amplicons in this study. The detailed experimental protocol is described in Supplementary Method.

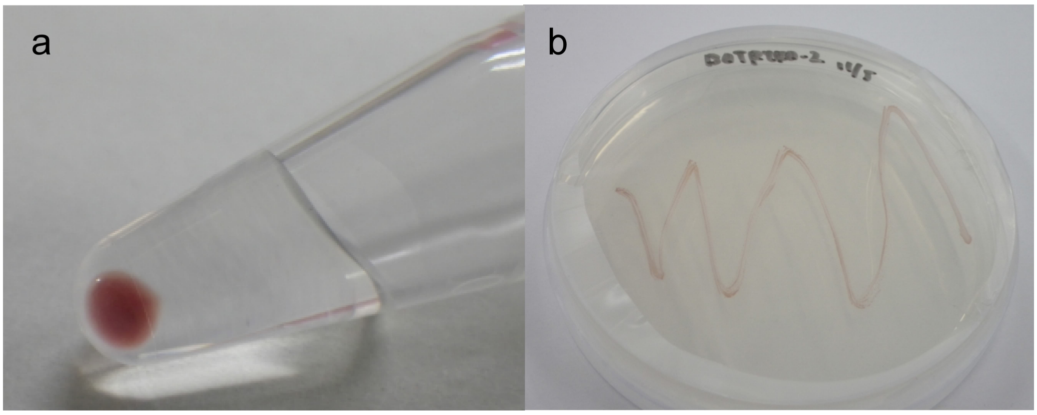

Figure S3

Visualization of BOTRYCO-2. **a**, a centrifuged GF/C filtrate of *B. braunii* Ba10$^{-/\mathrm{BOTRYCO\text{-}2}}$ in a 1.5-mL microtube indicating that BOTRYCO-2 is burgundy red in color. **b**, an axenic culture of BOTRYCO-2 on a plate.

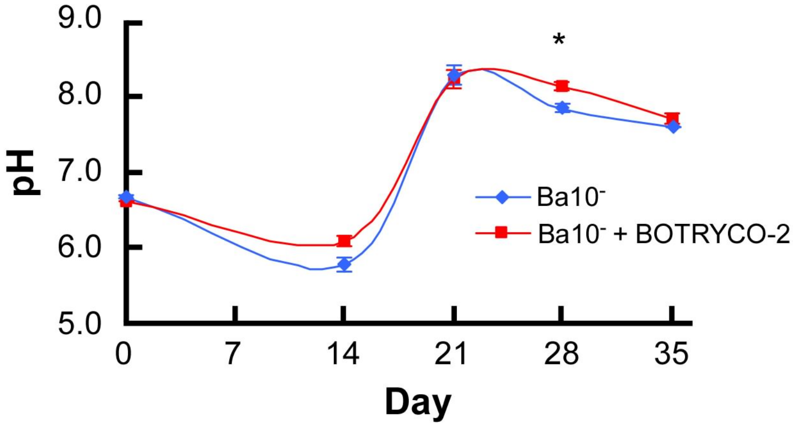

Figure S4

pH curves of the cultures indicated in Fig. 5. Bars indicate the standard error of four biological replicates. A significant difference between the two cultures was found only at day 28 (indicated by *) (homoscedastic one-tailed t-test, P = 0.0027). pH was measured using a LAQUAtwin pH meter (Horiba, Kyoto, Japan).

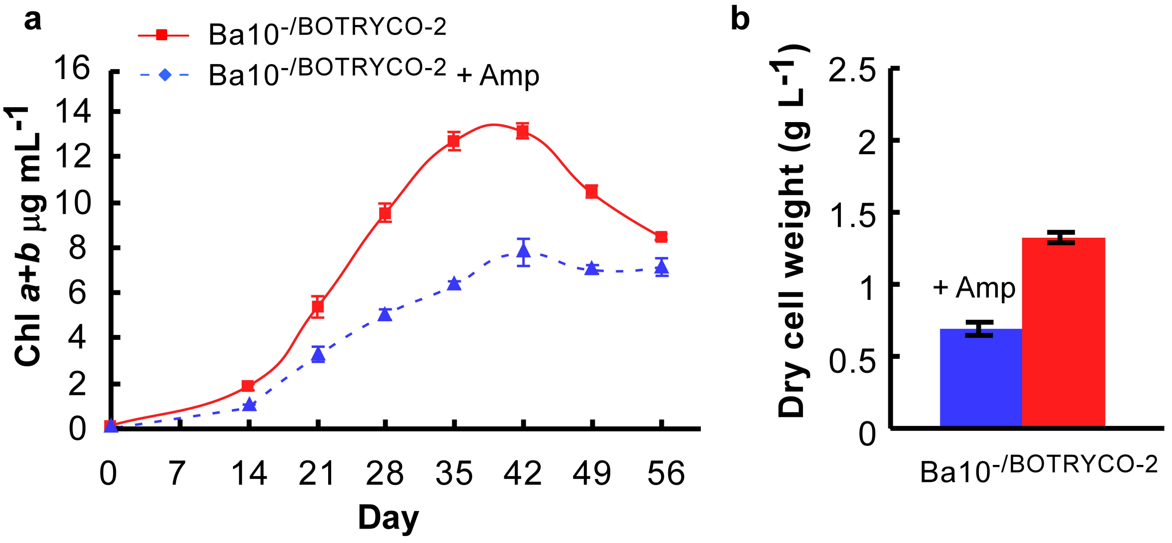

Figure S5

Growth and biomass of *B. braunii* Ba10$^{-/BOTRYCO-2}$. **a**, Growth curves of Ba10$^{-/BOTRYCO-2}$ with and without ampicillin treatment. Bars indicate the standard error of three biological replicates. Note that the chlorophyll concentration at day 56 did not match with the final biomass because of the breakdown of chlorophylls under possible nitrogen depletion (Hortensteiner & Feller, 2002). **b** Final biomass concentrations of *B. braunii* Ba10$^{-/BOTRYCO-2}$ (at day 56) on the basis of the growth experiments depicted in **a**. The y-axis indicate dry cell weight (dcw) per culture volume. Bars indicate the standard error of three biological replicates. The dcw of *B. braunii* Ba10$^{-/BOTRYCO-2}$ was significantly higher than that of ampicillin-treated cultures (homoscedastic one-tailed t-test, P = 0.00027).

Reference

Hörtensteiner, S. & Feller, U. Nitrogen metabolism and remobilization during senescence. *J. Exp. Bot.*, **53**, 927–937 (2002).

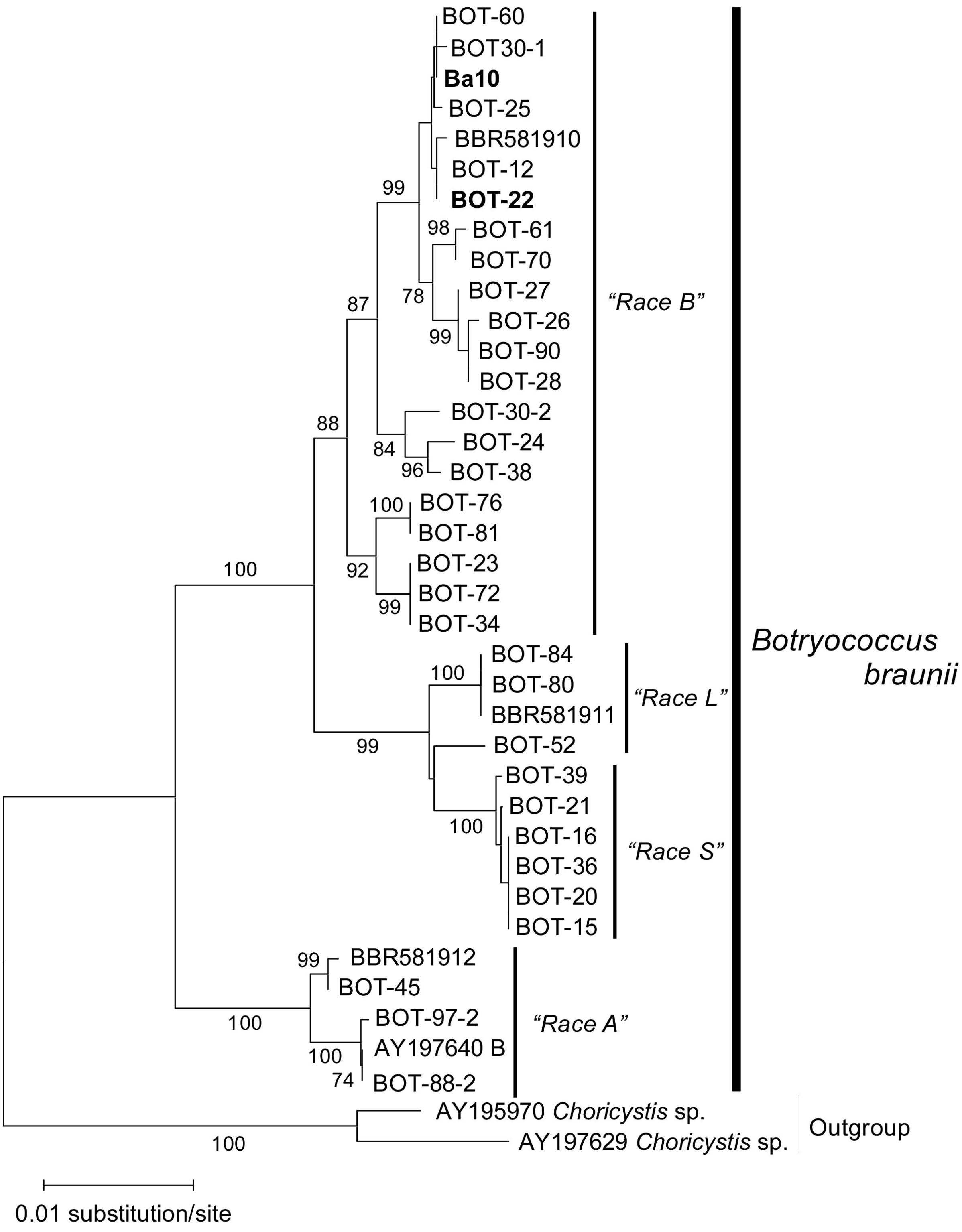

Figure S6

18S rDNA NJ phylogenetic tree of *B. braunii* on the basis of the alignment used in Kawachi et al (2012), plus the sequence of Ba10 (the final alignment consists of 1,524 bp without gaps). MEGA was used to construct the tree under the default setting. The strains used in this study are highlighted in boldface. The 18S rDNA sequence difference between Ba10 and BOT22 is 4 bp. Bootstrap values (>70) on the basis of

1,000 replicates are indicated at the respective nodes. Note that *B. braunii* strains are classified into races A, B, L, and S on the basis of (27) and Kawachi *et al*. (2012). The genomic DNA of Ba10 was extracted using the FastDNA SPIN kit. The 18S rDNA sequence of Ba10 was determined following the published protocol (Kawachi *et al.*, 2012). The sequence has been deposited in DDBJ under the accession number AB901105.

Reference

Kawachi, M., Tanoi, T., Demura, M., Kaya, K. & Watanabe, M. M. Relationship between hydrocarbons and molecular phylogeny of *Botryococcus braunii*. *Algal Res.* **1**,114–119 (2012).

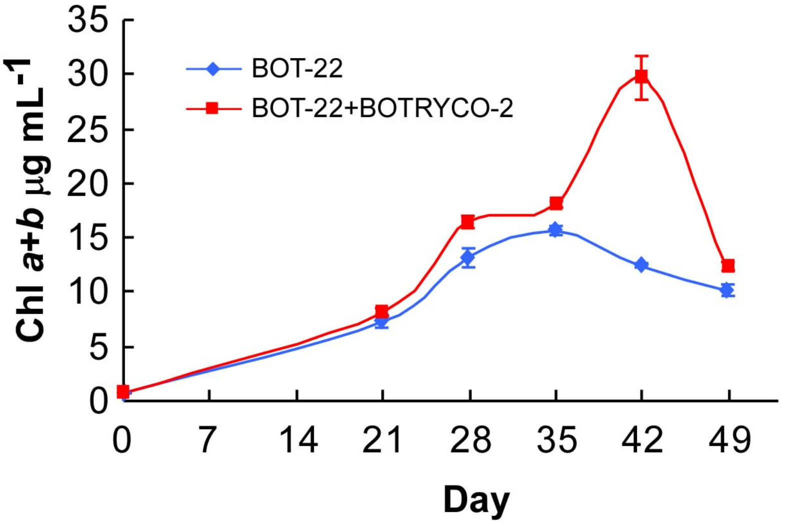

Figure S7

Growth curves of *B. braunii* BOT-22 inoculated with or without BOTRYCO-2. Bars indicate the standard error of five biological replicates. As shown in Supplementary Fig. S5, the final chlorophyll concentration did not match with the final biomass (Fig. 6).

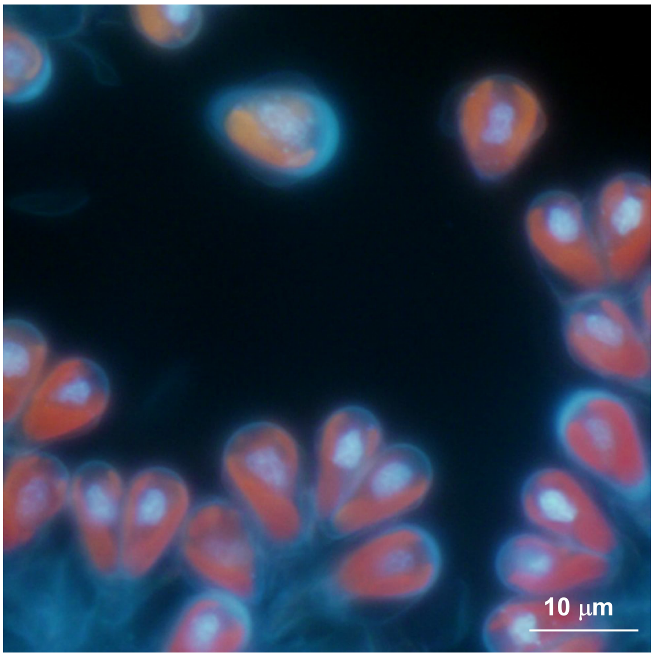

Figure S8

Fluorescent image of *B. braunii* Ba10$^-$ stained with DAPI. Spotted blue signals were visible in the nuclei of Ba10$^-$, whereas bacterial signals were absent in the image. Note that the extracellular matrix was slightly stained with DAPI, which can be discriminated from true signals by its lower fluorescent intensity.

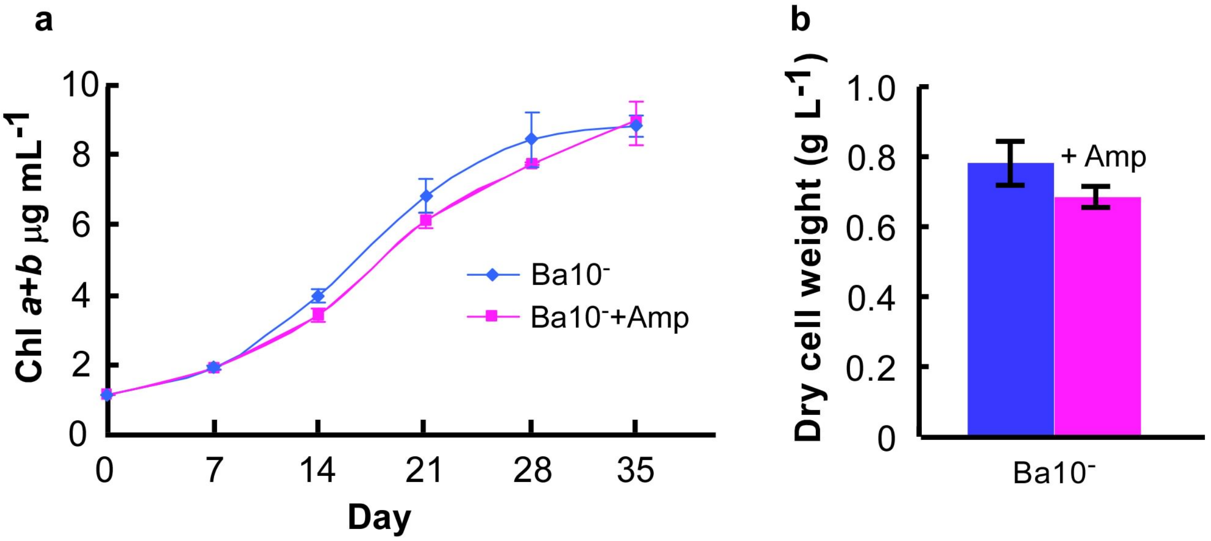

Figure S9

Effect of ampicillin treatment on the growth and biomass of *B. braunii* Ba10¯. **a**, growth curves of Ba10¯ inoculated with or without ampicillin. Bars indicate the standard error of three biological replicates. **b**, final biomass concentrations of *B. braunii* Ba10¯ (at day 35) on the basis of the growth experiments depicted in **a**. The y-axis indicate dry cell weight (dcw) per culture volume. Bars indicate the standard error of three biological replicates. The difference in dcws of *B. braunii* Ba10¯ was not significant between cultures with and without ampicillin treatment (homoscedastic two-tailed t-test, P = 0.279).

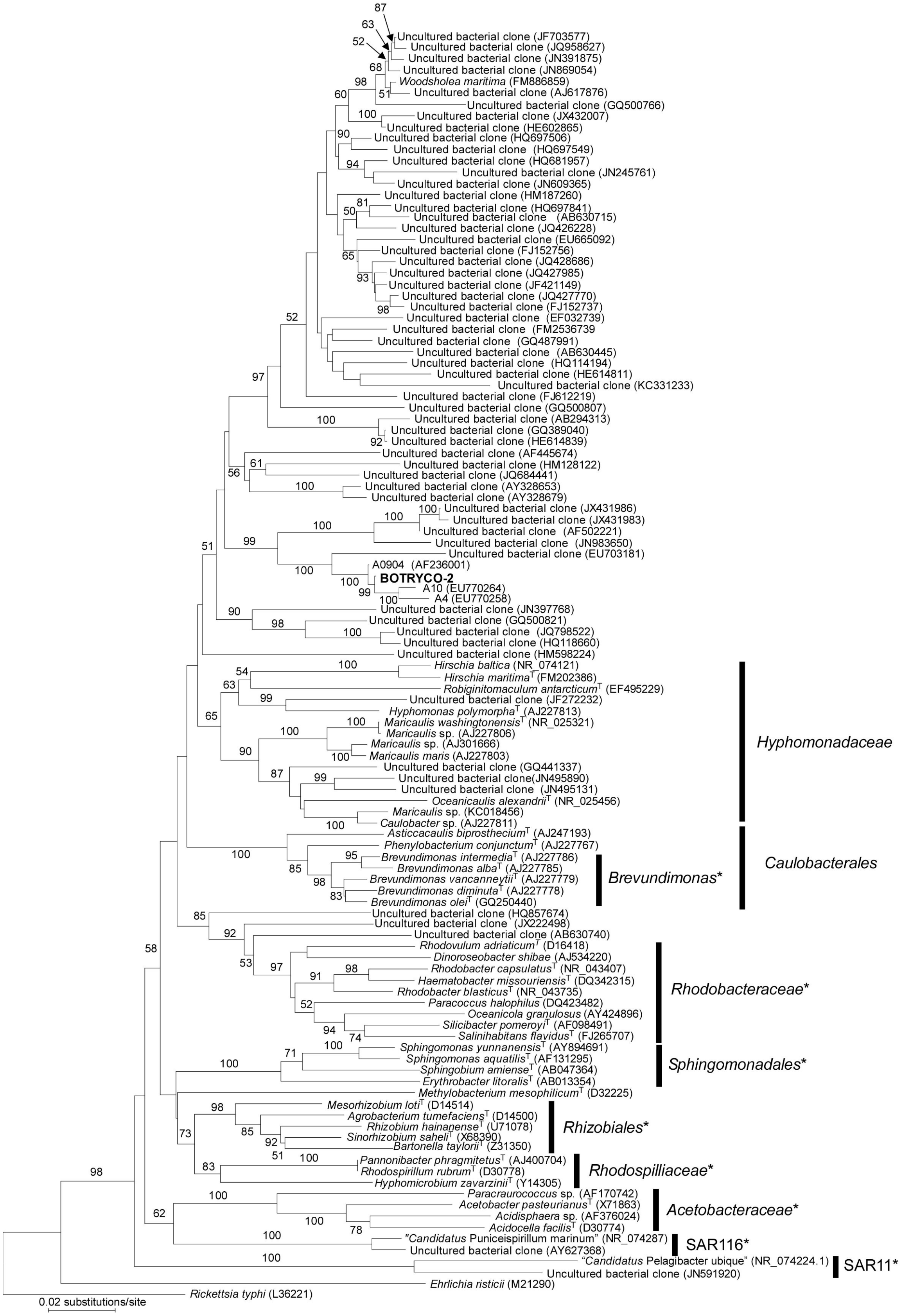

Figure S10

The full version of the NJ tree depicted in Fig. 3. Bootstrap values for each node (>50%) are indicated. Higher order taxa compressed in Fig. 3 are indicated with *.

Supplementary Table S1 Result of the PCR survey for BOTRYCO-2-related bacteria.

Locality	Latitude/Longitude	dd/mm/yy	PCR[b]	Polymorphism[a] 121	307	526
***M. aeruginosa* water bloom sample[c]**						
Tsuchiura, Lake Kasumigaura, Japan	N36° 4’ 46", E140° 12’ 32’’	01/09/04	+	A	A/C	G/T
Tsuchiura, Lake Kasumigaura, Japan	N36° 4’ 46", E140° 12’ 32’’	13/08/09	+		ND	
Tsuchiura, Lake Kasumigaura, Japan	N36° 4’ 46", E140° 12’ 32’’	27/08/09	+	A	C	T
Tsuchiura, Lake Kasumigaura, Japan	N36° 4’ 46", E140° 12’ 32’’	18/07/10	-	-	-	-
Tsuchiura, Lake Kasumigaura, Japan	N36° 4’ 46", E140° 12’ 32’’	01/08/10	+	A	A/C	G/T
Tsuchiura, Lake Kasumigaura, Japan	N36° 4’ 46", E140° 12’ 32’’	11/06/11	+	A/G	C	G/T
Tsuchiura, Lake Kasumigaura, Japan	N36° 4’ 46", E140° 12’ 32’’	18/06/11	+	A	C	T
Tsuchiura, Lake Kasumigaura, Japan	N36° 4’ 46", E140° 12’ 32’’	09/07/11	+	A	A	G
Tsuchiura, Lake Kasumigaura, Japan	N36° 4’ 46", E140° 12’ 32’’	18/07/11	+	A	A/C	G/T
Tsuchiura, Lake Kasumigaura, Japan	N36° 4’ 46", E140° 12’ 32’’	24/07/11	+		ND	
Tsuchiura, Lake Kasumigaura, Japan	N36° 4’ 46", E140° 12’ 32’’	31/07/11	+		ND	
Tsuchiura, Lake Kasumigaura, Japan	N36° 4’ 46", E140° 12’ 32’’	06/08/11	+	A	A/C	G/T
Tsuchiura, Lake Kasumigaura, Japan	N36° 4’ 46", E140° 12’ 32’’	12/08/11	+	A	A/C	G/T
Tsuchiura, Lake Kasumigaura, Japan	N36° 4’ 46", E140° 12’ 32’’	21/08/11	+	A	A/C	T
Tsuchiura, Lake Kasumigaura, Japan	N36° 4’ 46", E140° 12’ 32’’	28/08/11	+	A	A/C	T
Tsuchiura, Lake Kasumigaura, Japan	N36° 4’ 46", E140° 12’ 32’’	06/09/11	+	A	A/C	G/T
Tsuchiura, Lake Kasumigaura, Japan	N36° 4’ 46", E140° 12’ 32’’	13/09/11	+		ND	
Tsuchiura, Lake Kasumigaura, Japan	N36° 4’ 46", E140° 12’ 32’’	18/09/11	+	A	A/C	G/T
Tsuchiura, Lake Kasumigaura, Japan	N36° 4’ 46", E140° 12’ 32’’	26/09/11	+	A	C	T
Tsuchiura, Lake Kasumigaura, Japan	N36° 4’ 46", E140° 12’ 32’’	03/10/11	+	A	A/C	T
Yasuzuka, Lake Kitaura, Japan	N36° 7’ 19", E140° 31’ 56’’	15/08/10	+		ND	
Yasuzuka, Lake Kitaura, Japan	N36° 7’ 19", E140° 31’ 56’’	05/09/10	-	-	-	-
Rokko Oh-hashi, Lake Kitaura, Japan	N36° 5’ 8", E140° 32’ 18’’	24/07/10	+		ND	
Lake Senba, Japan	N36° 22' 7.2", E140° 27' 42.8"	21/08/10	+		ND	
Lake Senba, Japan	N36° 22' 7.2", E140° 27' 42.8"	06/09/10	+	A	C	T
Lake Senba, Japan	N36° 22' 7.2", E140° 27' 42.8"	22/09/10	+	A	A/C	G/T
Mizube Park, Lake Suwa, Japan[d]	N36° 03' 52.9", E138° 05' 22"	23/10/09	+	A/G	C	G/T
Kohan Park, Lake Suwa, Japan	N36° 02' 53", E138° 06' 39"	23/10/09	+	A/G	C	G/T
Funato Bridge, Lake Suwa, Japan	N36° 02' 14.7", E138° 05' 30.3"	23/10/09	-	-	-	-
Kamaguchi-Suimon, Lake Suwa, Japan	N36° 03' 13.1", E138° 03' 14.8"	23/10/09	+	A/G	C	G/T
Kamaguchi-Suimon, Lake Suwa, Japan	N36° 03' 13.1", E138° 03' 14.8"	11/09/10	+	A/G	C	G/T
Lake Kandawgyi, Myanmar	N16° 47’ 40.2", E96° 10’ 10.9’’	17/05/10	+		ND	
Lake Tonle Sap ST1, Cambodia	N13° 13’ 39.1", E103° 49’ 14.9’’	01/03/11	+	A	A/C	G/T
Lake Tonle Sap ST2, Cambodia	N13° 12’ 5.3", E103° 49’ 35.0’’	01/03/11	+	A	A/C	G/T
Lake Tonle Sap ST3, Cambodia	N13° 12’ 43.5", E103° 53’ 59.5’’	01/03/11	+	A	A/C	T
Lake Tonle Sap ST4, Cambodia	N13° 10’ 25.2", E103° 59’ 31.3’’	01/03/11	-	-	-	-
Lake Tonle Sap ST5, Cambodia	N13° 07’ 16", E104° 03’ 28’’	01/03/11	+		ND	
Lake Tonle Sap ST7, Cambodia	N13° 13’ 21.5", E103° 47’ 24.3’’	02/03/11	+	A	A/C	G/T
Lake Victoria, Kenya[d]	Unspecified	23/08/10	+	A	C	T
***B. braunii* environmental sample**						
Sokobaru Dam, Japan[e]	Unspecified	19/07/14	+	A	A	G
**Strain**	**GenBank Acc. No.**					
BOTRYCO-2[d]	AB900796			A	A	G
A4[d]	EU770264			A	A	G
A10[d]	EU770258			A	C	T

Footnotes:

[a] DNA polymorphism within the 27F/Ba1R amplified fragment. ND, not determined.

[b] Results of BOTRYCO-2 specific PCR detection using the primer pair 27F/Ba1R.

[c] Bloom samples including >10^5 cell mL^{-1} of *M. aeruginosa* were analysed.

[d] Samples or strains whose seqences are indicated in the alignment of Supplementary Fig. S2.

[e] *B. braunii* was collected using a plankton net and a centrifuged pellet of *B. braunii* cells was used for PCR. *M. aeruginosa* was not detected in the sample under a microscope.

Supplementary Methods

Establishment of an axenic culture of *B. braunii* Ba10

An axenic culture of *B. braunii* Ba10 ($Ba10^{-}$) was established from the original culture of Ba10 (with coexisting bacteria). Ba10 was cultured in 20 mL of liquid AF-6 medium in a 25-ml test tube for 2 months with ampicillin (final concentration, 50 μg L^{-1}) under the same temperature, light and CO_2 conditions as in the culture experiments described in the main text. Next, a small colony (approximately 0.5 mm) was picked using a micropipette in a sterile chamber and was washed several times by repeated transfer to the fresh liquid AF-6 in a microtube using the micropipette. Finally, the colony was transferred to the liquid AF-6 medium and was incubated for 3 months under the same culture conditions as described above. Axenicity of the culture was confirmed by microscopic observation of the cells with DAPI staining (Supplementary Fig. S9) and negative results for PCR detection of 16S rDNA using the bacterial universal primer pair 27F/1492R (50) and the specific primer pair 27F/Ba1R (Fig. S2). The axenicity of $Ba10^{-}$ was further confirmed by the experiment in Fig. 5, showing that no bacterial signal was found in the GF/C filtrates of $Ba10^{-}$ at every sampling point.

16S rDNA analysis

20 μL of *B. braunii* $Ba10^{-/BOTRYCO-2}$ culture was incubated at 97 °C for 5 min,

and 1 μL of this heat-treated solution was used as a PCR template. Using ExTaq DNA polymerase (Takara, Shiga, Japan), the nearly full-length 16S rDNA was PCR amplified (25-μL volumes) using the bacterial universal primer pair 27F/1492R. Cycling parameters comprised an initial denaturation step of 94 °C for 3 min, followed by 40 cycles of 94 °C for 1 min, 55 °C for 1 min, and 72 °C for 2 min. PCR products were purified using ExoSAP-IT®† (USB Corp., Cleveland, OH) and were sequenced in both directions using a BigDye Terminator v1.1 Cycle Sequencing Kit (Life Technologies, Carlsbad, CA) with an Applied Biosystems 3130 Genetic Analyzer (Life Technologies). In addition to the primers 27F and 1492R, primers 533F (Weisburg et al, 1991) and 536R (Mummey & Stahl, 2004) were used for sequencing. To obtain the 5′ and 3′ ends of the PCR-amplified segment, the same PCR reactions were performed using the high fidelity Taq enzyme *Pyrobest* (Takara, Shiga, Japan), and the amplicons were cloned using the Zero Blunt® TOPO® PCR Cloning for sequencing kit (Life Technologies). Using the 27F primer, the 5′ regions of >20 clones were partially sequenced to confirm that only a single sequence was recovered from each culture (i.e., to confirm that only one bacterial species/strain was present in each culture). To avoid a potential PCR error, five clones were sequenced and compared with the sequence obtained by the direct sequencing experiment.

For phylogenetic analyses, 250 16S rDNA sequences similar to those of the

Ba10 symbiont were obtained from the NCBI DNA databank on the basis of the result of a BLAST search. Sequences representing related alphaproteobacterial species were also retrieved and included in the phylogenetic analyses. On the basis of a preliminary phylogenetic analysis, several sequences in phylogenetically defined clades that are distantly related to BOTRYCO-2 were excluded from subsequent analyses to minimize the number of OTUs while maintaining the recognized overall phylogeny of *Alphaproteobacteria*. In addition, all but one OTU showing an identical sequence were removed. An initial alignment was obtained using Clustal X version 1.8 (Thompson et al, 1997). This was checked visually and corrected manually, followed by the removal of ambiguous regions, yielding a final dataset consisting of 1,440 nucleotide sites (including gaps) of 113 OTUs.

The detailed methods for the NJ tree reconstruction and its statistical analyses are described (57). Prior to the ML tree reconstruction, all gaps were removed from the alignment and three OTUs that showed the same sequence as the other OTUs were removed, yielding a dataset consisting of 1,206 nucleotide sites of 110 OTUs. An ML tree reconstruction using RAxML was performed at Cipress Science Gateway (Miller et al, 2010) under the default settings, except that bootstrap analysis was performed with 1,000 replicates.

References

Miller, M. A., Pfeiffer, W. & Schwartz, T. Creating the CIPRES Science Gateway for inference of large phylogenetic trees in *Proceedings of the Gateway Computing Environments Workshop (GCE), 14 Nov. 2010, New Orleans,* 1–8. (2010).

Mummey, D. L. & Stahl, P. D. Analysis of soil whole-and inner-microaggregate bacterial communities. *Microbial Ecol.* **48**, 41–50 (2004).

Thompson, J. D., Gibson, T. J., Plewniak, F., Jeanmougin, F. & Higgins, D.G. The CLUSTAL_X windows interface: flexible strategies for multiple sequence alignment aided by quality analysis tools. *Nucleic Acids Res.* **25**, 4876–4882 (1997).

Weisburg, W. G., Barns, S. M., Pelletier, D. A. & Lane, D. J. 16S ribosomal DNA amplification for phylogenetic study. *J. Bacteriol.* **173**, 697–703 (1991).
